# Multi-pronged approach to human mesenchymal stromal cells senescence quantification with a focus on label-free methods

**DOI:** 10.1038/s41598-020-79831-9

**Published:** 2021-01-13

**Authors:** Weichao Zhai, Jerome Tan, Tobias Russell, Sixun Chen, Dennis McGonagle, May Win Naing, Derrick Yong, Elena Jones

**Affiliations:** 1 Bioprocessing Technology Institute, A*STAR, 20 Biopolis Way, Centros, 06-01 Singapore; 2 Leeds Institute of Rheumatic and Musculoskeletal Medicine, Leeds, UK; 3 Singapore Institute of Manufacturing Technology, A*STAR, 2 Fusionopolis Way, Innovis, 08-04 Singapore

**Keywords:** Biological fluorescence, Ageing, Mesenchymal stem cells, Biophysics, Stem cells, Biological techniques, Optical spectroscopy

## Abstract

Human mesenchymal stromal cells (hMSCs) have demonstrated, in various preclinical settings, consistent ability in promoting tissue healing and improving outcomes in animal disease models. However, translation from the preclinical model into clinical practice has proven to be considerably more difficult. One key challenge being the inability to perform in situ assessment of the hMSCs in continuous culture, where the accumulation of the senescent cells impairs the culture’s viability, differentiation potential and ultimately leads to reduced therapeutic efficacies. Histochemical $\upbeta$-galactosidase staining is the current standard for measuring hMSC senescence, but this method is destructive and not label-free. In this study, we have investigated alternatives in quantification of hMSCs senescence, which included flow cytometry methods that are based on a combination of cell size measurements and fluorescence detection of SA-$\upbeta$-galactosidase activity using the fluorogenic substrate, C${_{12}}$FDG; and autofluorescence methods that measure fluorescence output from endogenous fluorophores including lipopigments. For identification of senescent cells in the hMSC batches produced, the non-destructive and label-free methods could be a better way forward as they involve minimum manipulations of the cells of interest, increasing the final output of the therapeutic-grade hMSC cultures. In this work, we have grown hMSC cultures over a period of 7 months and compared early and senescent hMSC passages using the advanced flow cytometry and autofluorescence methods, which were benchmarked with the current standard in $\upbeta$-galactosidase staining. Both the advanced methods demonstrated statistically significant values, (r = 0.76, p $\le$ 0.001 for the fluorogenic C${_{12}}$FDG method, and r = 0.72, p $\le$ 0.05 for the forward scatter method), and good fold difference ranges (1.120–4.436 for total autofluorescence mean and 1.082–6.362 for lipopigment autofluorescence mean) between early and senescent passage hMSCs. Our autofluroescence imaging and spectra decomposition platform offers additional benefit in label-free characterisation of senescent hMSC cells and could be further developed for adoption for future in situ cellular senescence evaluation by the cell manufacturers.

## Introduction

Owing to human mesenchymal stromal cells’ (hMSCs) multipotent differentiation potential, trophic functions and applications in cell and gene therapy^1^, they have attracted considerable research and clinical interests. In 2006, the International Society for Cellular Therapy (ISCT) proposed a set of minimal criteria to characterize MSCs including cell surface marker expression (must express CD105, CD73 and CD90, and lack expression of CD45, CD34, CD14 or CD11b, CD79$\upalpha$ or CD19 and HLA-DR surface molecules), must be plastic-adherent when maintained in standard culture conditions, and must be able to differentiate into osteoblasts, adipocytes and chondroblasts in vitro^2^. Based on their high proliferative potential in vitro, hMSCs have been applied extensively in cell-based therapy of graft-versus-host disease, liver failure and rejection after liver transplant, multiple sclerosis and myocardial infarction^3,4^. However, variable outcomes of hMSCs transplantation were observed, resulting from difficulties in controlling the fate of transplanted cells^5^ to reduced therapeutic efficacy after transplantation. Despite the cultures’ compliance to the ISCT definitions, these variable outcomes can be attributed by the different hMSCs tissue sources used^6,7^, through the different expansion procedures and the use of different media. On top of these factors, replicative senescence^8^ of hMSCs also significantly impact on the therapeutic efficacy, but is not currently included in MSCs release criteria for their therapeutic use^9^. Therefore, assessing hMSCs cultures for the presence of senescent cells remains critically important but is not routinely performed in hMSCs manufacturing processes^10^.

Numerous studies have documented that in hMSCs cultures, the amount of highly proliferative cells declines as the passage number increases, resulting in late passages of large and almost non-proliferative senescent hMSCs^11–13^. The study by Wagner et al. on serially passaged hMSCs revealed gradual changes in the global gene and miRNA expression^12^. Their study concluded that these senescent-link changes in gene and protein expression were not only associated with senescent passages, but also observed at the start of in vitro expansion. Thus, this accumulation of senescent cells from early passages suggests that identifying senescent cells in early hMSCs cultures can be an important analytical step to ensure the best hMSCs product quality for cell-based therapy.

Though MSCs have been actively applied in industry clinical trials of allogeneic transplantations^14^, there is still much to understand about MSCs aging and replicative senescence status to improve the long-term safety and efficacy of MSCs engraftment. At the molecular level, retinoblastoma protein (Rb) or p53 pathways triggers the cellular senescence process^15^. Additionally, senescent cells typically exhibit senescence-associated $\upbeta$-galatosidase (SA-$\upbeta$-gal) activities, acquire persistent DNA damage nuclear foci (PDDF) that contain DDR proteins ($\upgamma$ H2AX and 53BP1)^16^ and secrete growth factors, proteases and cytokines, which some of these biological features can be explained by the senescence-associated secretory phenotype (SASP). The ISCT criteria released in 2006^2^ is the current standard for regulatory approvals on hMSCs, but factors such as cellular senescence are not included in this standard criteria. Human MSCs attain replicative senescence, a feature likely to occur in industrial-scale MSC expansions, that impair their ability to suppress inflammation and reduce their therapeutic efficacy. Other clinical release criteria include microbiology testing and potency assessments^10^.

**Figure 1:**
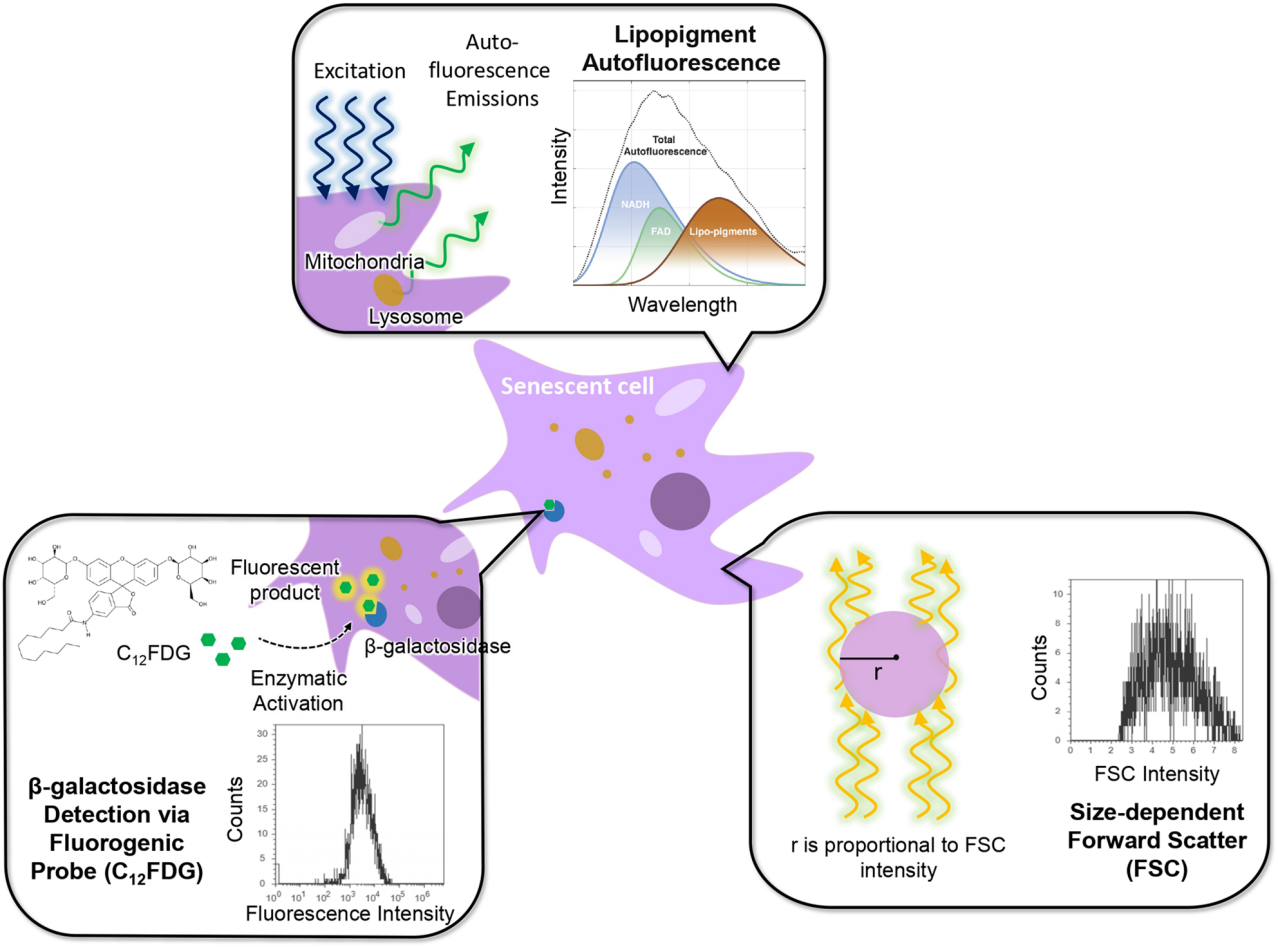
Various methods used for automatic quantification of hMSCs senescence in this study. Top: New autofluorescence method that employs cell lasing through endogenous fluorophores to collect autofluorescence signals. Bottom left: fluorescence-based detection of $\upbeta$-galactosidase activities using fluorogenic substrate C$_{12}$FDG through enzymatic activation and flow cytometry analysis. Bottom right: flow cytometry forward scatter (FSC) measurements for hMSCs cell size determination. All three flow cytometer and autofluorescence methods in quantification of hMSCs senescence were benchmarked with the cytochemical $\upbeta$-galactosidase staining method.

Among the various methods in characterising senescent hMSCs, the detection of senescence-associated $\upbeta$-galactosidase (SA-$\upbeta$-gal) activity through staining at the optimal lysosomal pH^17,18^ is the most contemporary standard. This method is closely associated with the accumulation of senescent cells, can be easily applied and detected at near-neutral pH, and was tested as a useful biomarker for detection of senescence in culture and even in vivo in rodents and primates^19^. The SA-$\upbeta$-gal serves as a positive control for other senescence characterization methods being developed, and can be more conclusive in senescence characterization in combination with flow cytometry or automated image analysis methods^20^. The detection of higher SA-$\upbeta$-gal activity is associated with an increase in lysosomal mass and accumulation of increased levels of *GLB1* mRNA and protein in senescent cells^17,21^. Cytochemical staining of SA-$\upbeta$-gal is one way to quantify the level of cell senescence as the percentage of senescent cells can be determined through counting the number of blue stained cells in the total population^22^. However, this method is time consuming and subjective. In contrast to this cytochemical method, a fluorescence-based method (Fig. 1 bottom left) differentiates between senescent and non-senescent cell populations more accurately based on incubation with C${_{12}}$FDG, a fluorogenic substrate for $\upbeta$-galactosidase, as it becomes fluorescent after entering the cell and cleaved by the enzyme^23^. Furthermore, the flow cytometric method provides cell size measurement of hMSCs through forward scatter (FSC)^24^ (Fig. 1 bottom right) and potentially offers a high-throughput alternative to the cytochemical method to quantitatively evaluate hMSCs senescence. This is linked to senescent cells generally displaying flattened and enlarged cell size^1^, which can be measured on the FSC channel^24^.

In our previous work, a method based on acquiring and processing native signals from live cells using the label-free technique of autofluorescence spectroscopy has been developed^25^. Autofluorescence methods have found many applications in biomedical research and diagnosis^26^. Its signal comes from a unique class of autofluorescent bio-molecules native to cells^27^, and these bio-molecules can be differentiated based on the specific spectral distribution of their autofluorescence emissions. Of particular interest among these bio-molecules are lipofuscin and lipofuscin-like pigments^28^, and their correlation with the state of senescence. Lipofuscin is formed by lipids, metals and misfolded proteins, which is especially abundant in nerve cells, cardiac muscle cells and skin^29^. Lipofuscin-like pigments are distinguished from lipofuscins and are bipartite granules consisting of an autofluorescent electron-dense pigment and electron-lucent lipid components^30^. Both lipofuscins and lipofuscin-like pigment give similar autofluorescence properties and will be measured collectively and referred to as lipopigments in our study. In contrast to the current standard in the detection of SA-$\upbeta$-gal activity, which is either cytochemical^31^ or fluorescence-based^22^, the autofluorescence spectroscopy method is non-destructive and label-free. Furthermore, the amount of emission corresponds to the biomolecule quantities^25^, and in theory, can be used to directly determine the extent of the cellular ageing process and senescent status. One challenge of the previously reported autofluorescence method is that it is still challenging in acquiring autofluorescence due to its weak intensities^32^.

Therefore, the aim of this work was to carry out MSCs senescence characterization through various methods including flow cytometry methods such as cytochemical staining through C$_{12}$FDG and forwards scatter, and our recently developed label-free autofluorescence spectroscopy method^25^ for measuring hMSCs senescence (Fig 1 top). For this purpose, autofluorescence results were compared with the flow cytometer FSC and C$_{12}$FDG measurements, and later compared with the $\upbeta$-galactosidase staining results.

## Results

### hMSCs characterisation

**Figure 2:**
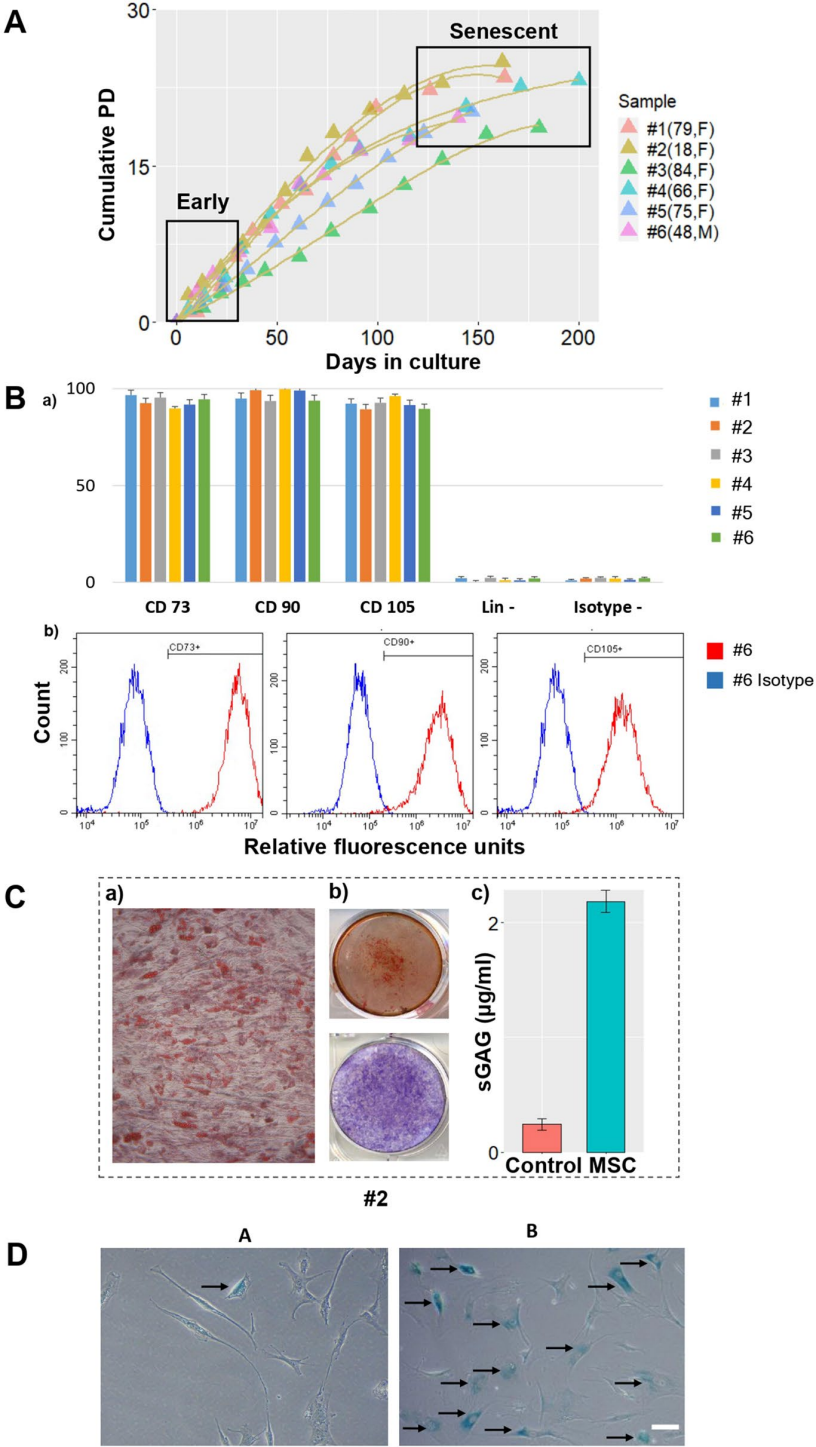
Characterisation of hMSCs used in this study. (**A**) Cumulative population doubling of the six selected donor samples (biological repeats n = 6) collected through in vitro expansion over a period of 7 months. Early passage cells are defined when their cumulative PD value is closest to six and senescent passage cells are defined as cells failed to double in 2 weeks’ time. (**B**): (a) Combined data on ISCT phenotypic characterization of all six donor MSC samples on CD73, CD90, CD105, Lin-: lineage negative, Isotype-: isotype control antibodies. Bars represent mean values and error bars represent standard deviations (SDs) (b) Representative histograms for donor #6 on CD73, CD90 and CD105 characterization. (**C**) Representative hMSCs differentiation results (donor #2) from left to right: (a) Oil Red O stained lipid vesicles after adipogenesis (b) Alizarin red staining demonstrating calcium deposition (middle upper) and alkaline phosphatase (middle lower) both indicating osteogenic differentiation (c) hMSCs chondrogenesis with sGAG levels higher than a control sample of hMSCs not cultured in chongrogenic media. (**D**) $\upbeta$-galactosidase staining images of early (**A**) and senescent passages (**B**) of a selected donor sample. At least 200 cells were measured and counted per $\upbeta$-galactosidase staining experiment. Arrows indicate $\upbeta$-galactosidase positive hMSCs. Scale bar indicates 50 $\upmu \text {m}$.

To prepare a hMSCs cell bank at different passages for hMSC senescence characterisation, cells from six donors were cultured for 7 months. Then their cumulative population doublings (cPD) averages for early passage cell (cPD = 6.13) and senescent passage cell (cPD = 20.6) were plotted against days in culture (Fig. 2A). Early passage cells (E) were defined as having cPDs below six^33^ and senescent passage cells (S) are defined as cell achieved less than 1 PD in 7 days^34^. It is interesting to note that hMSCs from the youngest donor (18,F) displayed the highest growth rate but the growth curve also coincided with that of the ageing donor (79,F). Though the data displayed a general trend that the growth rate plateaued as hMSCs approached senescence, no further inference could be made between the growth rate of hMSCs and donor age, or time to senescence, based on the growth curves alone.

As clinical outcomes of hMSCs may vary due to the different sources of the hMSCs harvested, the different expansion procedures and the usage of different media, standard characterisation of hMSCs at early passages is performed to confirm the hMSCs nature of manufactured cells. The bone marrow derived hMSCs (BM-hMSCs) used in our experiments were first characterised using ISCT recommended criteria and protocols detailed in the method section and results are summarized in Fig. 2B. Consistent with the minimal criteria to define hMSCs^2^, the cells collected and harvested through in vitro expansion expressed CD105, C73 and CD90, and lacked the expression of CD45, CD34, CD14, CD19 and HLA-DR (indicated as lineage-negative, Lin-), and can be further employed for hMSCs senescence studies.

Furthermore, in vitro expanded hMSCs (donor #2) demonstrated tri-lineage differentiation potential as required by the ISCT criteria^2^ (Fig. 2C). Oil Red O staining showed that hMSCs underwent adipogenesis to form adipocytes with the generation of lipid vesicles (Fig. 2Ca). Alizarin red staining of hMSCs after osteogenesis revealed the presence of calcium, and combined with alkaline phosphatase staining results, indicated a successful osteogenesis process (Fig. 2Cb). Furthermore, hMSCs were capable of chondrogenesis with levels of sulphated glycoaminoglycans (sGAGs) elevated as compared to hMSCs that were not cultured in a chondrogenic media (Fig. 2Cc).

$\upbeta$-galactosidase staining was next performed on early and senescent passages for all six donor samples with selected staining images shown in Fig. 2D. The senescent passage cells generally displayed a flattened and enlarged morphology as compared to the spindle-like shape of the early passage cells (Fig. 2D). Percentage of $\upbeta$-galactosidase positive stained cells were computed for both early and senescent passage cells, showing a fold difference range (1.757–3) and statistically significant p value (p $\le$ 0.001) between E and S passages (Supplementary materials Table 1). $\upbeta$- galactosidase results were further employed as the benchmark for evaluation of the flow cytometer and autofluorescence methods in senescent hMSCs quantification.

### Assessing early and senescent passage cells using autofluorescence and flow cytometry methods

**Figure 3:**
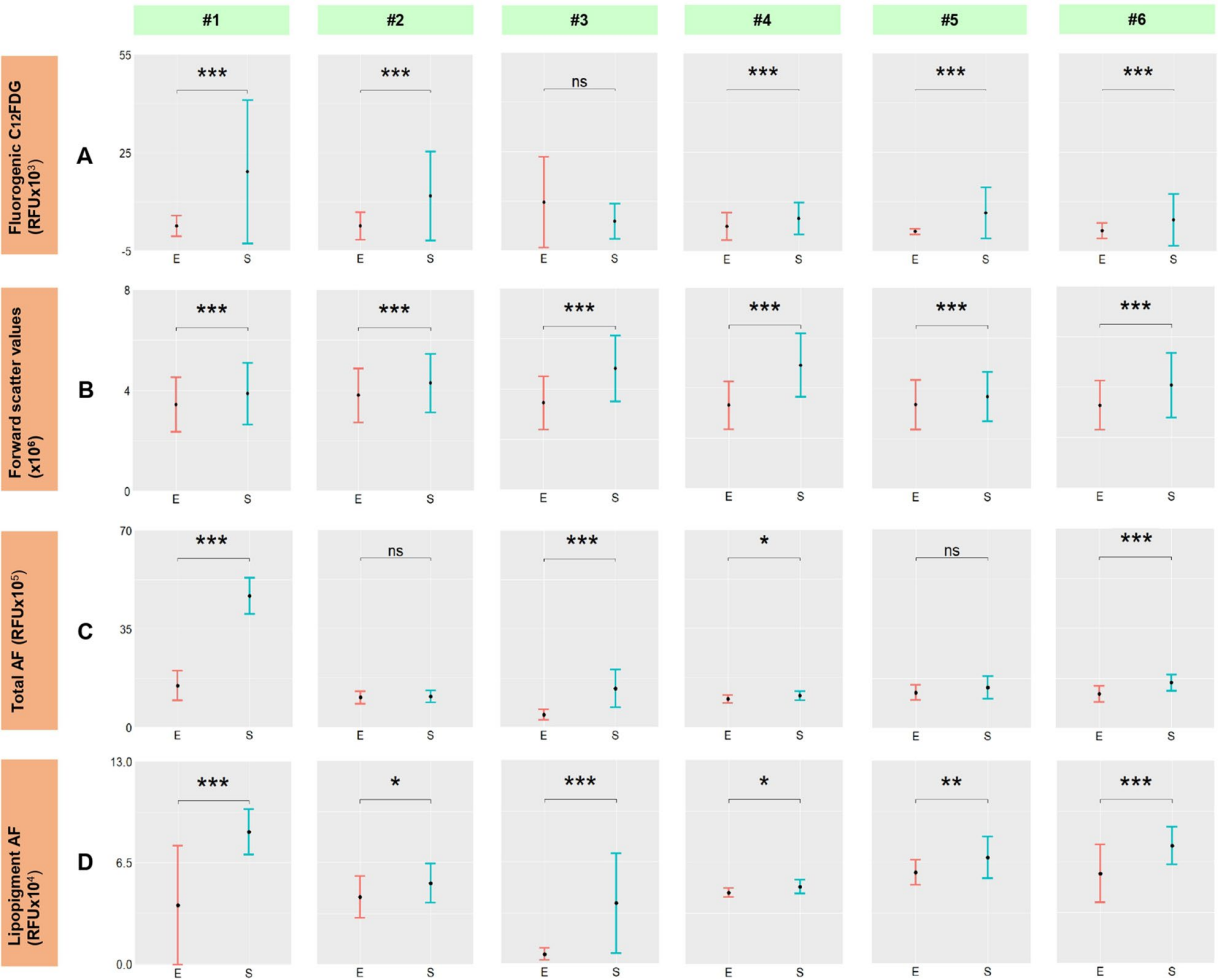
Error bar plots showing comparisons between the early and senescent hMSCs passages for six donors on (**A**) fluorescent-based $\upbeta$-galactosidase staining through C${_{12}}$FDG mean (**B**) forward scatter mean (**C**) total autofluorescence mean (**D**) lipopigment autofluorescence mean. Error bar plots were produced based on at least 10 cells measured per sample (experimental repeats n = 10) and 100 cells involved per autofluorescence run, and $10^5$ cells per flow cytometry measurement. Statistical analysis: one-tailed unequal variance t-test was performed between early and senescent data. Pairs with significant differences are marked with ***(P $\le$ 0.001), **(P $\le$ 0.01), *(P $\le$ 0.05) and ns indicates no significance (P > 0.05).

The fluorescent-based $\upbeta$-galactosidase staining through the fluorogenic substrate C${_{12}}$FDG results demonstrated statistical significance for five out of the six donor samples between early and senescent passage cells as shown in Fig 3A. On top of analysing the lysosomal activities for senescent hMSCs characterisation, hMSC cell size measurements were also employed for identifying senescence in culture^35^ based on the quantitative and high throughput data acquisition power of the flow cytometer. The forward scatter measurements through flow cytometry analysis displayed statistically significant results (p $\le$ 0.001) across all six donor samples and demonstrated high confidence in classification of early and senescent passage cells (Fig. 3B). Increases in senescent MSC cell sizes were confirmed using cytospin preparations followed by measurements of individual cells’ areas with a fold difference range (1.531–2.937) (Supplementary materials Table 2, Figures 1 and 2).

To evaluate the autofluorescence method in quantification of hMSCs senescence, the total autofluorescence intensity (Fig. 3C) and the autofluorescence contribution from lipopigments (Fig. 3D) were compared across the six donor samples between early and senescent passages, and one-tail unequal variance t-tests were performed. Fluorescence output from lipopigments, showing varying degrees of statistical significance for all of the six donor samples (Fig. 3D), demonstrated higher confidence in distinguishing between early and senescent cells than the total autofluorescence intensity measurements.

### Comparing autofluorescence and flow cytometry results with $\upbeta$-galactosidase staining results

**Figure 4:**
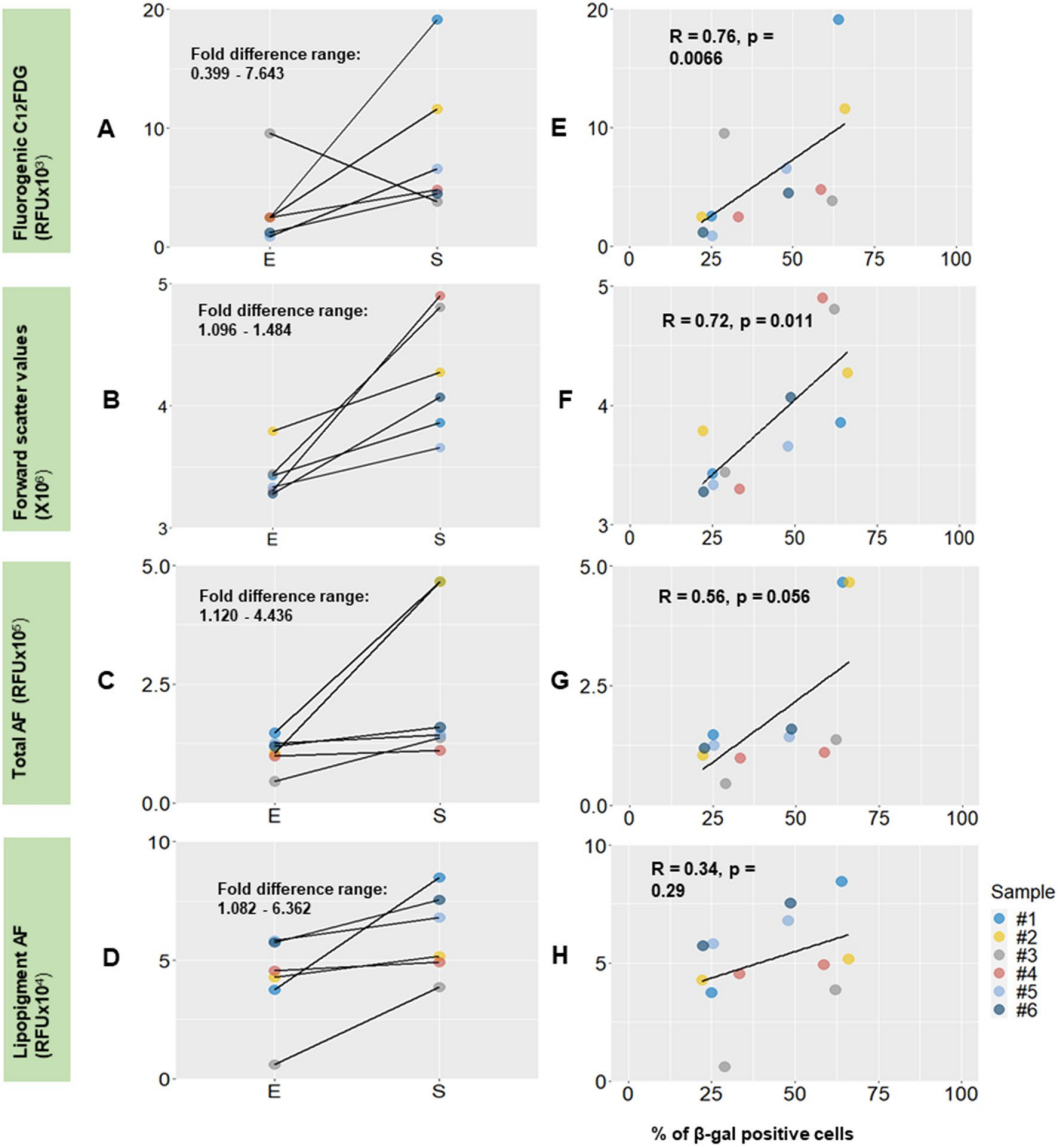
Fold difference (**A**–**D**) and correlation (**E**–**H**) plots of the various senescence quantification methods of hMSCs benchmarking with the $\upbeta$-galactosidase staining method. (**A**) fluorescent-based $\upbeta$-galactosidase staining through C${_{12}}$FDG mean (**B**) forward scatter mean (**C**) total autofluorescence mean (**D**) lipopigment autofluorescence mean. E—early, S—senescent passages. Spearman’s correlation coefficient, a statistical measure of the strength of a monotonic relationship between paired data, was employed to benchmark the various autofluorescence and flow cytometry methods with $\upbeta$-galactosidase.

The fold difference plots (Fig. 4A) indicated that the C${_{12}}$FDG method generated the highest fold difference value from the range (0.399–7.463). The autofluorescence methods also output distinguishable fold difference ranges for total autofluorescence (1.120–4.436) and for autofluorescence contribution from lipopigment (1.082–6.362) to differentiate between early and senescent passage cells. Furthermore, though autofluorescence measurements do not provide as good fold difference range as by the C${_{12}}$FDG method, their results are more consistent in predicting the direction of change from early to senescent passages (Fig. 4C,D) with no data showing opposite trend as seen in C${_{12}}$FDG method (Fig. 4A).

To further evaluate the potential of flow cytometry and autofluorescence methods as new advanced methods in senescent hMSCs characterisation, results graphically displayed in Fig. 3 were compared with the current standard using $\upbeta$-galactosidase staining. The below analysis is required as a full growth of cells for several months before characterisation is difficult to achieve by general cell manufacturers and in situ senescence evaluation is thus needed. By benchmarking with $\upbeta$-galactosidase staining, we found that the flow cytometric C${_{12}}$FDG staining and forward scatter methods were significantly correlated with $\upbeta$-galactosidase (p < 0.05). The spearman correlation value of the C${_{12}}$FDG and FSC flow cytometric methods also suggested that the results were biologically significant (R > 0.7) (Fig. 4E,F). Thus, the proportion of $\upbeta$-galactosidase positive, senescent hMSCs, can be potentially predicted based on data presented above. The data points for the total autofluorescence and autofluorescence from lipopigments quantification methods were more scattered giving lower r and non-significant p values (Fig. 4G,H), which could be resulted by the lower number of cells analysed.

## Discussion

### Comparison of the advanced methods with the current standard in $\upbeta$-galactosidase staining

To assess the autofluorescence method as non-destructive alternative to the current standard in the detection of SA-$\upbeta$-gal activity through cytochemical staining, we compared the total autofluorescence mean between early and senescence passage donor cells. The total autofluorescence output distinguished four out of six donor cell samples and generated a fold difference range (1.120–4.436) between E and S passage cells (Fig. 4C), suggesting good sensitivity in quantification of senescent hMSC. As expected, senescent hMSCs displayed well defined morphological changes in our study, demonstrating flattened and enlarged morphology as shown in our $\upbeta$-galactosidase staining images (Fig. 2D), due to an excess of actin fibers and cell debris^1^. Bertolo et al. similarly reported a positive relationship between hMSC cell size and cellular autofluorescence^36^ resulted by an increase in the fluorescent cellular organelles (i.e. mitochondria and lysosomes). These studies and correlations explained our observation that senescent hMSCs displayed a higher total autofluorescence output (Fig. 3C). In Bertolo’s study, the autofluorescence signal was collected using CytoFLEX flow cytometer with excitation laser at 488 nm and detection optic at 525/50 nm range^36^. Based on our autofluorescence spectra, detection at 525/50 nm range corresponds to autofluorescence contribution from flavin adenine dinucleotide (FAD) and does not encompass the full autofluorescence range for lipopigment, which ranges between 450 and 700 nm^37^. Thus, we further decoupled and analysed the autofluorescence contribution from lipopigment and its association with cellular senescence.

Results from the lipopigment autofluorescence between early and senescent passage cells demonstrated significant differences between all six pairs of E and S passage cells, with a good fold difference range (1.082–6.362) but it showed large variation between cultures. Lipopigment is the important indicator of cell senescence^28,29^, and contributes to an increase level of autofluorescence in senescent hMSCs^1^. From previous cell studies, proliferative cells dilute the deposits of the lipopigment during cell division^29^, showing low or no accumulation of the pigment. Conversely, non-proliferative cells result in lipopigment accumulation in the lysosomes and cell cytoplasm as lipopigment cannot be degraded due to its polymeric and highly cross-linked nature^29^. Thus, a higher lipopigment autofluorescence intensity suggests the accumulation of the pigment, indicating the cells reaching a non-proliferative stage and becoming senescent. In the recent studies reported by Feng et al.^38^, autofluorescence intensity positively correlated with cell senescence in retinal pigment epithelial (RPE) cells that demonstrated similar potential of the method in identification of cellular senescence. Overall, both the total autofluoresence mean and lipopigment autofluorescence methods demonstrated promising potential in senescent hMSCs characterisation.

The flow cytometry assisted detection of senescent hMSCs through C${_{12}}$FDG and FSC measurements are rapid, high-throughput methods as compared to the cytochemical staining of $\upbeta$-galactosidase, which is labour intensive and results are operator dependent. Out of the six donor samples analysed, the FSC results produced consistent, statistically highly significant p values (p $\le$ 0.001) between all early and senescent pairs (Fig. 3B), which the consistency could be attributed by the large sample size per measurement (n > 10$^5$) as compared to 10 cells per autofluorescence run. By benchmarking with $\upbeta$-galactosidase, the FSC method produced biologically significant spearman correlation values (R > 0.7) and p values (p $\le$ 0.05), demonstrating similar capability in senescent cells characterisation as $\upbeta$-galactosidase. Flow cytometry forward scatter measurements allow discrimination of cells by size as FSC intensity is proportional to the diameter of the cell, and resulted by light refraction within the cell. Majore et al. had first employed FSC as a standardized cell size measurement tool to identify subpopulation of MSC-like cultures from human umbilical cord^24^. A recent report from Oja et al. had suggested that cell area in correlation with cell size could be one of the most statistically significant parameters in representing the morphological changes that associates with biochemical and gene expression markers of senescent cells^20^. This result was further validated by Bertolo et al. finding that senescent cells generally display flattened and enlarged cell morphology as compared to the spindle-like form of the early passage cells^36^. These results correspond with our observation that the high throughput FSC method produced statistically significant results in distinguishing between early and senescent hMSCs, as well as with our cell size measurements using cytospins.

Though FSC method produced statistically highly significant p values in classification between early and senescent passage cells, the fold difference range for FSC measurements (1.096–1.484) did not reflect a clear distinction between the E and S pairs. Although Oja et al. reported that cell size could be one of the most statistically significant parameters revealing senescence induced morphological expansion of MSCs, cells were chosen from selected passages and plated on 2D surface for imaging and analysis using Cell Omics Morphology Explorer software^20^. This 2D measurement of cell size is in contrast to the FSC measurements carried out in our study where cells were measured in suspension. In fact, results from our cytospin 2D measurements of cell areas showed a fold difference range of (1.531–2.937) between E and S pairs (Supplementary materials Figure 2), close to the fold difference range provided by forward scatter measurements, and comparable with the fold differences measured by other methods.

### Comparison across the advanced methods

Despite the fact that flow cytometry assisted C${_{12}}$FDG method generated the highest spearman correlation and fold difference range (0.399–7.643) between early and senescent pairs, its results are highly sensitive to the assay and cell staining conditions. It is important to note that previous fluorescent detection of SA-$\upbeta$-gal activities has been performed mostly on fibroblasts^23,35^, and thus method optimization is required to ensure that the test is suitable for hMSCs senescence characterisation. In our experimental design, both the C${_{12}}$FDG staining time and concentration were carefully tuned and hMSCs were stained in monolayer conditions. Furthermore, a minimum of 10$^5$ cells were stained.

On top of being sensitive to assay conditions, the flow cytometry method presents several other limitations such as complex experimental procedure and extended preparation time. The 1 h C${_{12}}$FDG incubation time is considerably longer than the autofluorescence method where samples do not require prior incubation with specific marker before the measurement but significant improvement from the $\upbeta$-galactosidase staining method where samples need to be incubated overnight. Both the autofluorescence and $\upbeta$-galactosidase staining methods require additional cell adherent step prior to measurement but only $\upbeta$-galactosidase method involves staining and destructively labelling. The flow cytometry methods also destructively measure a minimum of 10$^5$ cells per run in suspension as compared to none of the cells being labelled per autofluorescence run. From the perspective of cell-based therapy, this substantial amount of stained cells is no longer fit for clinical or therapeutic purposes after the flow cytometry analysis.

In contrast to the flow cytometry methods, the autofluorescence methods demonstrated considerable potential in distinguishing between early and senescent passages. As discussed, similar results were also reported in literature, where fluorescence from lipopigment were employed and proven as feasible senescence indicators through fluorescence microscopy^29,38^ . The fold difference range between early and senescent pairs produced by both the total autofluorescence mean (1.120–4.436) and the lipopiment mean (1.082–6.362) were much higher than the fold difference range produced by FSC measurements (Fig. 4B), but comparable to the fold difference range of the C${_{12}}$FDG measurements (0.399–7.463). This suggests higher sensitivity of the label-free autofluorescence methods in identification of senescent hMSCs and its potential as alternatives to the $\upbeta$-galactosidase method. However, it is important also to note that the autofluroescence method has its limitation in the number of cells measured owing to the limited number of viable cells after seeding into the silicon wells.

Additionally, as compared to the flow cytometer instrument required for experiment, the autofluorescence method is more adoptable and economical. For the autofluorescence method, after the 24-h growth and attachment of hMSCs in the medical grade silicon well, only a simple swap of the imaging solution is required before individual autofluorescence measurements. Each autofluorescence run takes only 10 min to prepare and 2 s to measure as compared to the 1-h preparation time required for the fluorescence based $\upbeta$-galactosidase method. The spectrometer set-up does require routine calibration before a new set of experiments to ensure the maximum intensity output is collected by the fluorescence microscope. The key benefit of this method is that cells were only incubated in the imaging solution for a short period of time, and thus offers the possibility for the non-modified cells to be recycled for other downstream quality control processes in cell-based therapy.

Indeed rapid and high throughput, the flow cytometry method however often requires labelling with specific biomarkers for acceptable classification accuracy. Furthermore, there exists fundamental trade-off between throughput and accuracy in any measurement system^39^ as hydrodynamic focusing dilutes the cell suspension and reduces the throughput rate in order to bring cells into the focus plan of an optical system. Overall, the flow cytometry method is more suitable for large-scale cell analysis applications, where sacrificial cell samples can be provided for characterisation purposes only. When a small number of donor cells are available for expansion and characterisation, particularly for autologous applications^40^, the autofluorescence method would be better at identifying individual senescent cells without destructive labelling, maximising the final output of therapeutic-grade hMSC cultures. Overall, the spectra decomposition method offers rapid analysis of the autofluorescence output from cells, which could be conveniently adopted and applied to label-free measurements at the scale of hundreds of cells.

In our study, we recognise that the analysis was restricted to early and senescent passages on six donor samples. However, we used the same number of donors as were analysed in Oja et al. study^20^ and more stringent criteria for defining early and senescence MSC passages based on accrued population doubling compared to Bertolo et al. study^36^. Also, the various methods of senescence characterization were primarily performed on bone marrow MSCs owing to the fact that bone marrow derived hMSCs is one of the most common and longest utilized type of MSCs^41,42^. Recent studies on the same subject similarly focused on bone marrow MSCs^20,36^, and one study^36^ also assessed adipose-tissue derived stem cells (ADSCs), where similar findings to bone-marrow MSCs were found. To further validate our autofluorescence platform as the label-free alternative for MSCs characterization, MSCs from other tissue source should be further measured and evaluated based on the autofluorescence method.

Despite the advantages mentioned for autofluorescence methods, we should not disregard the inherent variability of cells (donor to donor variations) while evaluating the effectiveness of the different methods in hMSCs senescence characterization. In the future, autofluorescence methods should be compared to a broader range of methods used for MSC senescence assessment including gene expression of senescent markers (i.e. p16$^\text {INK4A}$ and CCND2)^36^ and qPCR-based method with single telomere length analysis (STELA)^43^. Furthermore, from literature, confluence and different media components are the factors to be considered to affect the autofluorescence signals^36^. In our study, we made sure that the media composition was consistent across all samples and the starting seeding concentration for autofluorescence samples remained the same; however, variability in the samples being measured due to cellular heterogeneity may still exist and is donor related^44^. In addition, the heterogeneity and auto-differentiation characteristic of MSC could have resulted in the differences in fold-changes (between early-passage and senescent MSCs) observed for different cultures. Interestingly, no strong correlations were found between autofluorescence measurements and adipo-, osteo- and chondrogenic differentiation of MSCs, as well as donor age and telomere length, in a similar recent study^36^ whereas the correlations with SASP proteins were much stronger. Future work is needed to determine whether lipopigment measurements described in the present study would provide notable advantages over total autofluorescence in terms of the assessment of MSC SASP phenotypes.

There is limited literature on using the lipopigment component to characterize MSCs senescence, and it is the next step we will pursue for the development and refining of our autofluorescence method. Staining of lipopigment^45^ is proposed as the next step to identify the sole autofluorescence contribution from lipopigment, and we will compare with the results obtained from our current study on using spectra decomposition method to analyse lipopigment autofluorescence.

## Conclusion

In this work, we evaluated the potential of the flow cytometry and autofluorescence methods in senescent hMSCs identification and benchmarked with the current standard in $\upbeta$-galactosidase staining. Autofluorescence was studied in two ways, namely via the total autofluorescence output and specific autofluorescence stemming from lipopigements. These label-free autofluorescence methods distinguished between early and senescent passage cells based on higher autofluorescence output from the endogenous fluorophores of senescent cells. Flow cytometry based forward scatter and fluorogenic substrate through C${_{12}}$FDG produced high throughput and accurate differentiation between early and senescent hMSCs. However, there exist limitations in these methods owing to the sensitivity of the flow cytometry method to assay conditions. In contrary, the autofluorescence alternative offers rapid and consistent measurements of the fluorescence output from cell organelles with no prior incubation or modification of cells required.

Though the autofluorescence method reported is not ready to be fully extended for on-line monitoring applications, it is a more adoptable and economical way of rapid assessment of senescent cells through the different stages of the manufacturing process. The spectra decomposition tools developed in house could be further extended to in situ live cell monitoring at a larger scale. Overall, our label-free semi-automated autofluorescence cell characterisation method has the potential to offer a wider scope of applications in hMSCs quality assessment and in monitoring of the cell therapy products during manufacturing.

## Methods

### hMSCs isolation and culturing

Sections of normal spinous process were collected from patients undergoing spinal surgery at Leeds General Infirmary for corrections of scoliosis or decompressions of the lumbar or thoracic vertebrae. Samples obtained from 6 patients (biological repeats n = 6 median age 75) were assigned sample numbers and bone fragments containing bone marrow. Unless otherwise stated, the reagents used were from Sigma Aldrich. Digestion mix containing collagenase was prepared as previously described^46^ were used to initiate hMSc cultures and bone samples were vortexed and incubated in 37 °C water bath for 4 h to extract hMSCs. The digestion mix containing extracted cells was poured through cell strainer into a fresh tube. The bone sample was repeatedly washed by sterile PBS to extract the remaining cells and solution again poured through strainer into the tube until PBS was clear after vortexing the sample. The solution containing cells was centrifuged at 700 rcf for 10 min at room temperature. The cell pellet was re-suspended in 1× red blood cell lysis buffer and incubated at room temperature for 5 min, centrifuged again at 700 rcf for 10 min and re-suspended in 10 ml of DMEM for cell counting.

Cells were seeded into T75 flask at 4000 per cm$^\text {2}$ and serially passaged through in vitro expansion with StemMACS media (Miltenyi Biotec) over 7 months. The seeding density of hMSCs for each expansion was at least 10$^5$ cells per T75 flask with a harvest of approximately 10$^6$ cells after passaging. At least 3 × 10$^5$ cells were frozen per passage per donor sample with freezing media (45% StemMACS, 45% FBS—fetal bovine serum, 10% DMSO) and stored as cell bank for later autofluorescence analysis. All our samples are regularly tested for mycoplasma (MycoAlert PLUS, Lonza) and any positive cultures are destroyed and not used in experiments. Population doubling and cumulative population doubling of each passage per donor sample were calculated, as previously described^47^, and the passage closest to a PD value of six was sent for MSC characterisation according to ISCT guidelines^2^.

### ISCT and isotype characterisation

Selected early and senescent passage cells were defrosted from frozen vials in a water bath at 37 °C. The cells were counted to be of at least $10^5$ cells per tube. The cell pellet was re-suspended in 200 $\upmu \text {l}$ of blocking buffer (0.5% BSA—bovine serum albumin, 2% FBS in 1× PBS) and incubated for 15 min at room temperature. FACS buffer (0.5% BSA, 0.05% Sodium Azide in 1× PBS) of 200 $\upmu \text {l}$ were added to the suspension and the solution was split into tubes with 50 $\upmu \text {l}$ each. Antibodies against MSC positive markers (Miltenyi Biotec): CD73-PE (Clone AD2), CD90-PerCP-Vio700 (Clone REA897), CD105-FITC (Clone 43A4E1) and negative markers (Viogreen): CD14 (Clone REA599), CD19 (Clone LT19), CD34 (Clone AC136), CD45 (Clone REA747), HLA-DR (Clone REA805) were added and solutions were incubated for 15 min at 4 °C in the dark. FACS buffer of 500 $\upmu \text {l}$ were added to each tube to wash off non-binding antibodies. Tubes of stained and unstained cells were spun down at 400 rcf for 5 min, re-suspended in 500 $\upmu \text {l}$ FACS buffer and the data were acquired by Attune Acoustic Focusing Flow Cytometer (Applied Biosystems). The FlowJo software (version 10.7) (http://www.flowjo.com/solutions/flowjo/downloads) was used for data analysis with debris excluded by gates, and the percentage of cell expressing these surface markers were also recorded.

### hMSCs differentiation studies

Minimally passaged (P $\le$ 3) hMSCs (donor #2) were assessed for tri-lineage potential in accordance with ISCT minimum criteria. Briefly, for adipogenesis 5 × $10^5$ cells/well were seeded into 24-well plates and cultured for 3-weeks in a complete adipogenic media containing: DMEM (Life Technologies), 10%FCS (Biosera), 10% horse serum (Stem Cell Technologies), 0.5 mM isobutylmethylxantine, $60\;\upmu \text {M}$ indomethacine (ICN) and 0.5 mM hydrocortisone. Wells had half media changes every 3 days. At day-21 cells were fixed with 10%-formalin before lipid vesicles were stained using an Oil Red O solution for 10 min after which wells were counter-stained with haematoxylin for 45 s. Samples were stored in PBS whilst imaged using Olympus CKX41 light microscope and an Olympus C-7070 camera.

Chondrogenesis was conducted in Eppendorf tubes seeded with 2.5 × $10^5$ hMSCs, which were centrifuged at 650 rcf for 5 min to pellet the cells and cultured in a chondrogenic media containing: high-glucose DMEM (Life Technologies), $200\;\upmu \text {M}$ ascorbic-2-phosphate, 1mM sodium pyruvate, $40\; \upmu \text {g/ml}$ proline, 1 mg/ml bovine serum albumin, 10 nM dexamethasone, 10 ng/ml TGF$\upbeta$3 (R&D Systems) and 1% ITS+. Samples were cultured for 3 weeks with half media change three times per week. On day 21 media was completely removed, and the pellet washed carefully twice with PBS. $100\;\upmu \text {L}$ of 1mg/ml papain solution was added and allowed to incubate overnight at 65 °C in the water bath as previously described^48^. After which the contents were mixed well and frozen at $-20$ °C until a glycosaminoglycan (GAG) was measured using Blyscan Glycosaminoglycan Assay (Bicolor) was performed as per manufacturer’s instructions.

Osteogenesis was assessed using either alizarin red or alkaline phosphatase staining. $10^4$ hMSCs were seeded into 12-well plates and cultured for 3-weeks with bi-weekly half media changes. Samples were cultured in an osteogenic media containing, DMEM (Life Technologies), 10% FCS (Biosera), $100\;\upmu \text {M}$ ascorbic-2-phosphate, 10 mM $\upbeta$-glycerophosphate and 100 nM dexamethasone.

Alkaline phosphatase staining was measured on the 14th day after initiation of osteogenesis, wells were fixed using a citrate/acetone solution, following fixation Fast-Blue solution was used as per manufacturers’ instructions. Alizarin red staining was assessed at day 21 after initiation of osteogenesis. Wells were fixed for 1 h in cold 70% ethanol. A 40 mM alizarin red aqueous solution was used to stain the cultures for 10 min at room temperature after which cultures were washed 3 times with distilled water. Both alkaline phosphatase and alizarin red stained wells were imaged using an Olympus CKX41 light microscope with an Olympus C-7070 camera attached.

### Cytochemical and flow cytometric detection of SA-$\upbeta$-galactosidase activities

To analyse the lysosomal activities of hMSCs for senescence characterisation using the cytochemical staining method, senescent cells histochemical staining kit was employed. Early and senescent passage cells were seeded in one well of a six-well plate with a maximum seeding density of $10^5$ cells per well to avoid confluence. After 24 h in a 37 °C incubator, growth medium was first aspirated from the early and senescent hMSCs. Cells were washed twice with 1 ml of PBS (Life Technologies). Fixation buffer of 1.5 ml was added per well and the cells were incubated for 6–7 min at room temperature. Cells were then rinsed 3 times with 1 ml of PBS. Staining mixture of 1 ml was next added per well. The plate was sealed with parafilm and incubated at 37 °C without $\text {CO}_{2}$ overnight. After incubation, the staining mixture was replaced with 1 ml of PBS. The blue-stained cells and the total number of cells were counted and the percentage of cells expressing $\upbeta$-galactosidase was calculated. At least 200 cells were counted in each well after staining.

For the fluorescence-based detection of $\upbeta$-galactosidase activities, selected early and senescent passages of at least $10^5$ cells were seeded per well of a six-well plate and placed in an incubator at 37 °C with 5% $\text {CO}_{2}$ for 24 h. $33\;\upmu \text {M}$ of $\text {C}_{12}\text {FDG}$ (Thermo Fisher) working solution was added to the designated wells in the six-well plate and incubated for 1 h. The working solution was then removed and the cell monolayer was washed twice with 1 ml PBS. The cells were harvested by trypsin followed by centrifuging at 600 rcf at 4 °C for 5 min. $\text {C}_{12}\text {FDG}$ fluorescence was acquired through FL1 channel on the Attune flow cytometer.

### Autofluorescence microspectroscopy

A previously reported autofluorescence microspectroscopy protocol for fibroblasts^25^ was adopted for hMSCs based on their similar adherent nature. Selected passages of hMSCs were seeded at a concentration of at least 3.0 $\times$ 10$^4$ cells/ml on glass coverslips (Schott) within square silicone wells fabricated from medical grade silicone (Wacker Chemie AG). These coverslips with cells in 1 ml of StemMACS media (Miltenyi Biotec) were then incubated in a CO$_{2}$ incubator for 24 h before autofluorescence microspectroscopy. Prior to measurements, the culture media was extracted and the remaining contents in the wells were washed twice with 1 ml of PBS. After washing, any excess PBS was remove and 100 $\upmu \text {l}$ of imaging solution (Thermo Fisher Scientific) was added. Five phase contrast images were obtained per coverslip at random locations through a 4$\times$ objective. Autofluorescence images and spectra were then taken through a 60$\times$ oil-immersion super apochromat objective (Olympus). At least 100 cells per early and senescent passage per donor were involved in each autofluorescence run. Autofluorescence output of at least 10 cells were recorded with measurements made at different locations within the silicon well (experimental repeats n = 10). This was followed by five background spectral measurements of a 100 $\upmu \text {l}$ volume of imaging solution placed on a clean region of the same coverslip.

### Autofluorescence spectral decomposition and data analysis

Autofluorescence spectra were similarly processed based on the previously reported method^25^. This was achieved via a MATLAB-based (version 9.5.0.94444 R2018b) (http://www.mathworks.com/products/compiler/matlab-runtime.html) software developed in-house that performed signal processing, background correction and spectral decomposition. Spectral decomposition involved a linear unmixing of the autofluorescence spectra into its constituent components. Each spectral component corresponded to a specific autofluorescent biochemical found natively in cells. In this study, four spectral components were used in the decomposition, namely nicotinamide adenine dinucleotide in both bound (NADH$_{bound}$) and free (NADH$_{free}$) forms, FAD and lipopigments^26^. Firstly, the software was trained to recognize the first three components’ emissions using reference solutions. NADH$_{free}$ was prepared by dissolving NADH in Tris buffer at pH 8.0 (BUF-1414-500ml-pH8.0, 1st Base, Singapore); NADH$_{bound}$ was prepared by mixing NADH$_{free}$ solutions with l-lactate dehydrogenase dissolved in the same Tris buffer; FAD in PBS without Ca$^{2+}$ and Mg$^{2+}$. Spectral fitting parameters for these components were compiled into library files. Subsequently, autofluorescence spectra for commercial senescent cells (ATCC, PCS-500-012) were processed with these three components and a fourth component that comprised estimations for lipopigments was generated. This thus trained the software to recognize lipopigments, and its spectral fitting parameters were similarly added to the library files. The compiled library files were then applied in the spectral decomposition of autofluorescence spectra from test hMSCs. For donor cell samples autofluorescence signal processing, the library obtained from commercial cell software training was applied with lipopigment identified as one of the four expected fluorophores. Both the fluorescent and background data were inputed for each early and senescent passage hMSCs per donor, and the wavelength range was set between 400 and 850 nm to encompass the full wavelength range of the selected fluorophores^26^. After signal processing, the decomposed autofluorescence spectra, the identified peak values for the fluorophores and the sum of intensities under each peak per measurement were stored and tabulated.

Autofluorescence mean and autofluorescecne contribution from lipopigment mean were then computed for statistical analysis. The unequal variance t-test which assumes that both groups of data are sampled from Gaussian populations, but does not assume those two populations have the same standard deviation, was employed to help to quantify the deviation between the means of the two measured parameters. Statistical analyses for the autofluorescence measurements were conducted using R (version 4.0.2) (cran.r-project.org/bin/windows/base/)^49^ with standard deviations computed through ggerrorplot^50^ and the standard deviations for the flow cytometry readings computed by the FlowJo software. Figures were produced using R studio packages ggplot2^51^ and ggpubr^50^.

## Ethics declarations

The study conducted is in accordance with the guidelines approved by North West-Greater Manchester West Research Ethics Committee (REC: 16/NW/0797) and Agency for Science, Technology and Research Institutional Review Board (IRB Reference: 2018-001). Patients gave written informed consent in accordance with the declaration of Helsinki.

## Supplementary Information

Supplementary Information.

## Data Availability

The datasets analysed during the current study are available from the corresponding author on reasonable request.
