# Supplementary material for: Multi-pronged approach to human mesenchymal stromal cells senescence quantification with a focus on label-free methods
Source: Sci Rep. 2021 Jan 13;11:1054. doi: 10.1038/s41598-020-79831-9 (PMC7807049; doi:10.1038/s41598-020-79831-9)
Supplement: Supplementary file 1 — Supplementary Information. [file 41598_2020_79831_MOESM1_ESM.pdf]

SUPPLEMENTARY INFORMATION

MULTI-PRONGED APPROACH TO HUMAN MESENCHYMAL STROMAL CELLS SENESCENCE QUANTIFICATION WITH A FOCUS ON LABEL-FREE METHODS

Weichao Zhai[1], Jerome Tan[1], Tobias Russell[2], Sixun Chen[1], Dennis McGonagle[2], May Win Naing[1,3], Derrick Yong[3*], and Elena Jones[2*]

[1]Bioprocessing Technology Institute, A*STAR, 20 Biopolis Way, Centros 06-01, Singapore

[2]Leeds Institute of Rheumatic and Musculoskeletal Medicine, Leeds, UK

[3]Singapore Institute of Manufacturing Technology, A*STAR, 2 Fusionopolis Way, Innovis 08-04, Singapore

[*]derrick-yong@simtech.a-star.edu.sg

[*]E.Jones@leeds.ac.uk

Table 1. Table of percentages of β-galactosidase positive early and senescent passage hMSCs with fold differences shown. At least 200 cells were counted per sample per passage. Statistical test produced significant p value ($p \leq 0.001$) between E and S passages. Statistical analysis: one-tailed unequal variance t-test was performed between early and senescent hMSCs staining data.

Sample	Percentage of early passage stained cells	Percentage of senescent passage stained cells	Fold difference
#1	25%	63.9%	2.556
#2	22%	66%	3
#3	28.9%	62%	2.145
#4	33.3%	58.5%	1.757
#5	25.3%	48%	1.897
#6	22.4%	48.7%	2.174

Table 2. Table of cytospin cell area measurements of early and senescent passages hMSCs of all patient MSCs samples with fold difference shown. At least 100 cells were measured per sample using ImageJ. Statistical test produced significant p value ($p \leq 0.01$) between E and S passages. Statistical analysis: one-tailed unequal variance t-test was performed between early and senescent hMSCs staining data.

Sample	Cell size of early passage cells/μm^2	Cell size of senescent passage cells/μm^2	Fold difference
#1	848.0	1338	1.578
#2	945.0	1447	1.531
#3	919.7	1430	1.555
#4	875.1	1845	2.108
#5	748.9	2093	2.795
#6	914.0	2684	2.937

Fig. 1. Cytospin images of early (A) and senescent (B) passages hMSCs from a representative patient sample. Circles marked out the examples of stained MSCs. Scale bar indicates 50µm.

A B

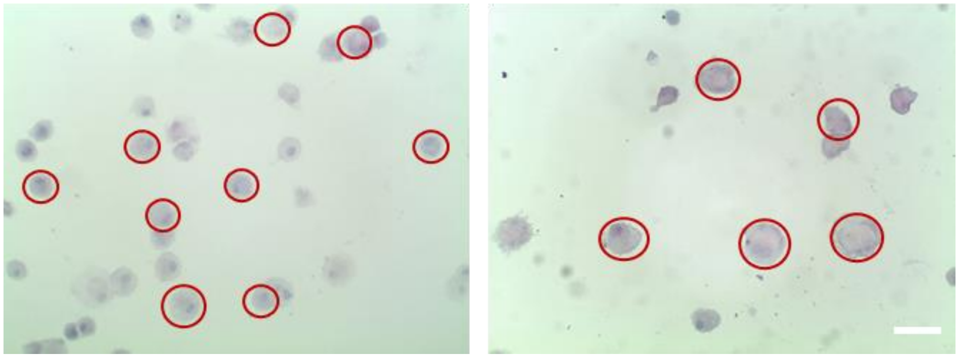

Fig. 2. (A) Fold difference and (B) correlation plots of the cytospin cell area measurements benchmarked with the β-galactosidase staining method. At least 100 cells were measured per cytospin sample. Spearman's correlation coefficient, a statistical measure of the strength of a monotonic relationship between paired data, was employed to benchmark the cytospin cell area results with β-galactosidase staining results with a significance value $p<0.05$. E – early, S – senescent.

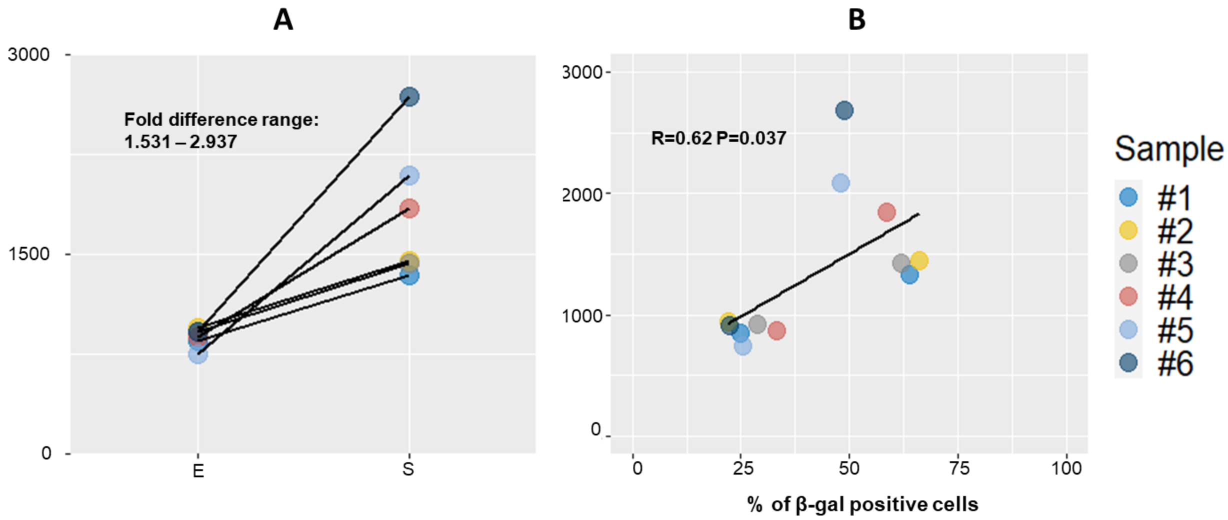

Supplementary method section on cytospin cell area measurements

For the cytospin cell area measurements, selected early and senescent passage cells of all six donor samples (n=6) of no more than 10^5 cells were trypsinized and re-suspended in 1ml of fresh DMEM. Pre-labelled glass slides for each early and senescent hMSC sample were mounted with a cuvette in the metal holder, and placed in a Cytospin 4 Cytocentrifuge (Thermo Fisher). 200µl of the cell suspension was loaded in each cuvette and was then spun down at 800rpm for 5 minutes. The cuvette was carefully detached from the slides and the area around cytocentrifuged cells was marked out with a marker. The slides were subsequently stained with Haematoxylin Eosin (H&E) stain with stained cell images taken by a microscope. Cell area were measured by Image J with at least 100 cells analysed per early and senescent passages per sample.
